# Supplementary material for: Role and mechanism of NCAPD3 in promoting malignant behaviors in gastric cancer
Source: Front Pharmacol. 2024 Apr 22;15:1341039. doi: 10.3389/fphar.2024.1341039 (PMC11070777; doi:10.3389/fphar.2024.1341039)
Supplement: Supplementary file 11 [file DataSheet2.ZIP › GSEA/Oncogenic signature/my_analysis.Gsea.1599461962066/MYC_UP.V1_UP.html]

Details for gene set MYC\_UP.V1\_UP[GSEA]

|  || Dataset | filtered\_dataset.sample\_info.cls#WT\_versus\_NCAPD3\_MUT |
| Phenotype | sample\_info.cls#WT\_versus\_NCAPD3\_MUT |
| Upregulated in class | WT |
| GeneSet | MYC\_UP.V1\_UP |
| Enrichment Score (ES) | 0.45783618 |
| Normalized Enrichment Score (NES) | 1.7292058 |
| Nominal p-value | 0.015517241 |
| FDR q-value | 0.12822308 |
| FWER p-Value | 0.634 |
Table: GSEA Results Summary

  

Fig 1: Enrichment plot: MYC\_UP.V1\_UP      
 Profile of the Running ES Score & Positions of GeneSet Members on the Rank Ordered List

  

| SYMBOL | TITLE | RANK IN GENE LIST | RANK METRIC SCORE | RUNNING ES | CORE ENRICHMENT || 1 | 153364 | MBLAC2 | 79 | 0.838 | 0.0458 | Yes |
| 2 | 90025 | UBE3D | 167 | 0.710 | 0.0702 | Yes |
| 3 | 51018 | RRP15 | 192 | 0.685 | 0.1367 | Yes |
| 4 | 6573 | SLC19A1 | 231 | 0.650 | 0.1889 | Yes |
| 5 | 57521 | RPTOR | 239 | 0.645 | 0.2627 | Yes |
| 6 | 91860 | CALML4 | 289 | 0.600 | 0.3009 | Yes |
| 7 | 84135 | UTP15 | 330 | 0.576 | 0.3426 | Yes |
| 8 | 54517 | PUS7 | 388 | 0.544 | 0.3682 | Yes |
| 9 | 56894 | AGPAT3 | 414 | 0.527 | 0.4147 | Yes |
| 10 | 10785 | WDR4 | 465 | 0.495 | 0.4393 | Yes |
| 11 | 83448 | PUS7L | 520 | 0.462 | 0.4571 | Yes |
| 12 | 51388 | NIP7 | 593 | 0.428 | 0.4578 | Yes |
| 13 | 1119 | CHKA | 670 | 0.390 | 0.4510 | No |
| 14 | 51154 | MRTO4 | 794 | 0.327 | 0.4028 | No |
| 15 | 51575 | ESF1 | 812 | 0.309 | 0.4284 | No |
Table: GSEA details [plain text format]

  

Fig 2: MYC\_UP.V1\_UP      
 Blue-Pink O' Gram in the Space of the Analyzed GeneSet

  

Fig 3: MYC\_UP.V1\_UP: Random ES distribution      
 Gene set null distribution of ES for **MYC\_UP.V1\_UP**
